# NeoAPACHE II. Relationship Between Radiographic Pulmonary Area and Pulmonary Hypertension, Mortality, and Hernia Recurrence in Newborns With CDH

**DOI:** 10.3389/fped.2021.692210

**Published:** 2021-07-12

**Authors:** Ilaria Amodeo, Nicola Pesenti, Genny Raffaeli, Francesco Macchini, Valentina Condò, Irene Borzani, Nicola Persico, Isabella Fabietti, Giulia Bischetti, Anna Maria Colli, Stefano Ghirardello, Silvana Gangi, Mariarosa Colnaghi, Fabio Mosca, Giacomo Cavallaro

**Affiliations:** ^1^Neonatal Intensive Care Unit, Fondazione IRCCS Ca' Granda Ospedale Maggiore Policlinico, Milan, Italy; ^2^Division of Biostatistics, Epidemiology and Public Health, Department of Statistics and Quantitative Methods, University of Milano-Bicocca, Milan, Italy; ^3^Department of Clinical Sciences and Community Health, Università degli Studi di Milano, Milan, Italy; ^4^Department of Pediatric Surgery, Fondazione IRCCS Ca' Granda Ospedale Maggiore Policlinico, Milan, Italy; ^5^Pediatric Radiology Unit, Fondazione IRCCS Ca' Granda Ospedale Maggiore Policlinico, Milan, Italy; ^6^Department of Obstetrics and Gynecology, Fondazione IRCCS Ca' Granda, Ospedale Maggiore Policlinico, Milan, Italy; ^7^Cardiology Department, Fondazione IRCCS Ca' Granda Ospedale Maggiore Policlinico, Milan, Italy; ^8^Neonatal Intensive Care Unit, Fondazione IRCCS Policlinico San Matteo, Pavia, Italy

**Keywords:** congenital diaphragmatic hernia, radiographic lung area, lung hypoplasia, pulmonary hypertension, mortality, recurrence of the hernia, FETO

## Abstract

Congenital diaphragmatic hernia is a rare disease with high mortality and morbidity due to pulmonary hypoplasia and pulmonary hypertension. The aim of the study is to investigate the relationship between radiographic lung area and systolic pulmonary artery pressure (sPAP) on the first day of life, mortality, and hernia recurrence during the first year of life in infants with a congenital diaphragmatic hernia (CDH). A retrospective data collection was performed on 77 CDH newborns. Echocardiographic sPAP value, deaths, and recurrence cases were recorded. Lung area was calculated by tracing the lung's perimeter, excluding mediastinal structures, and herniated organs, on the preoperative chest X-ray performed within 24 h after birth. Logistic and linear regression analyses were performed. Deceased infants showed lower areas and higher sPAP values. One square centimeter of rising in the total, ipsilateral, and contralateral area was associated with a 22, 43, and 24% reduction in mortality risk. sPAP values showed a decreasing trend after birth, with a maximum of 1.84 mmHg reduction per unitary increment in the ipsilateral area at birth. Recurrence patients showed lower areas, with recurrence risk decreasing by 14 and 29% per unit increment of the total and ipsilateral area. In CDH patients, low lung area at birth reflects impaired lung development and defect size, being associated with increased sPAP values, mortality, and recurrence risk.

**Clinical Trial Registration:** The manuscript is an exploratory secondary analysis of the trial registered at ClinicalTrials.gov with identifier NCT04396028.

## Introduction

Congenital diaphragmatic hernia (CDH) is a severe congenital malformation with a wide outcome variability (1). Pulmonary hypoplasia and persistent pulmonary hypertension (PH) represent the two main determinants of outcomes in patients, with still high mortality and morbidity (2–12). The radiographic assessment of the lung area has been proposed as an alternative method to evaluate pulmonary hypoplasia soon after birth (13–15). In newborns with CDH, lung area is correlated to the functional residual capacity measured through the diluted helium technique, and its increase is associated with tidal volume improvement in the first year of life (16, 17). The chest radiographic thoracic area (CRTA) was found to be lower in patients with poor prognosis and to predict survival to discharge from the Neonatal Intensive Care Unit (NICU) better than lung-to-head ratio at diagnosis (LHR) (18). However, a possible association between lung area and pulmonary hypertension has never been investigated.

Hernia recurrence represents one of the most common complications, and a large diaphragmatic defect is one of the main independent risk factors (19–25). The recurrence could occur weeks, months, or even years after the primary surgery, and patients often remain asymptomatic for a long time or until complications arise. Therefore, the overall risk of recurrence during the lifespan remains unknown (8). To our knowledge, an association between lung area and hernia recurrence has never been reported so far.

Since lung hypoplasia and vascular development are strictly related, our hypothesis was that lower lung areas at birth could determine higher mortality and higher pulmonary artery pressure (25–27). Moreover, we supposed that low lung area could indirectly reflect a large diaphragmatic defect size and be therefore associated with hernia recurrence (16, 18).

## Methods

The present study was carried out in accordance with the principles of good clinical practice and the Helsinki Declaration, as well as the national legislative and administrative provisions in force. This study was approved by the local Ethics Committee (Milan Area 2, Italy) with approval number OSMAMI-04/05/2020-0015998-U. Due to the retrospective nature of the study, the informed consent was waived by the Ethics Committee.

This study represents an exploratory secondary analysis of a previous retrospective cohort study called Assessment of the Pulmonary Area in Newborns with Congenital diaphragmatic HErnia (NeoAPACHE), performed at NICU of Fondazione IRCCS Ca' Granda Ospedale Maggiore Policlinico, Milan, Italy, on CDH patients over a 6-year period (January 2012–December 2018). A comprehensive description of the main study design has been previously published (NeoAPACHE I) (17).

In NeoAPACHE II, we aimed to evaluate the relationship between radiographic pulmonary area assessed on the first day of life and:

1. Pulmonary hypertension at birth, indirectly estimated by measuring the sPAP through tricuspid valve regurgitation gradient with echocardiogram;
2. Death during the first year of life; and
3. Recurrence of CDH among survivors at 1 year of life.

Moreover, the radiological features and outcomes of neonates candidate to FETO procedure were described and compared with those of the expectantly managed patients. At our Center, FETO was offered to fetuses with severe lung hypoplasia, defined as an O/E LHR <25 and <45% in left and right CDH, respectively, in the absence of major associated malformations and/or genetic anomalies known to have a significant impact on postnatal survival (28, 29).

### Subjects

All CDH patients admitted to our NICU are managed according to the CDH EURO Consortium Consensus guidelines (30). As previously described, we enrolled all newborns having a preoperative chest X-ray performed within 24 h after birth at our NICU. Death within 1 h, rotated, and air leak radiographs were excluded (17). Surgery was performed as soon as the patient achieved the hemodynamic and respiratory stability through median laparotomy with either primary repair or Gore-Tex^®^ patch insertion. According to CDHSG defect size classification, patching was performed in case of large defects (type C or D) (30–34). After discharge, all patients were included in a multidisciplinary follow-up program. In our Unit, we prefer to perform a chest X-ray at all time points during the first 2 years of life, then annually, aiming to detect asymptomatic recurrences early (8, 30, 35).

### Assessment of Radiographic Pulmonary Area

Each patient's pulmonary area was assessed by freehand tracing of the diaphragm and rib cage's perimeter, excluding the mediastinal structures and herniated organs (17). If the anatomy was particularly disrupted, only the aerated portion of the lung was considered. The corresponding area was automatically calculated by the software Synapse PACS (FUJIFILM Medical Systems USA, Inc.). On each radiogram, three measurements were performed:

1. Ipsilateral pulmonary area (cm^2^);
2. Contralateral pulmonary area (cm^2^); and
3. Total pulmonary area (cm^2^), obtained as the sum of the preceding two.

### Data Collection

Data regarding prenatal history, clinical, and surgical course were collected from each patient's electronic medical records. Hernia severity was defined according to the combined evaluation of observed/expected lung-to-head ratio (O/E LHR%), liver herniation, and side of the diaphragmatic defect (left, right, bilateral) (2, 28, 29). Echocardiograms performed after birth (T0), pre-surgery (T1), post-surgery (T2), and 7 days after surgery (T3) were reviewed, and reported sPAP values were recorded. CDH recurrence after surgical repair and the number of deaths in the first year of life were considered. Data acquisition was anonymous.

### Statistical Analysis

Continuous variables were reported as mean (standard deviation) or median (interquartile range); categorical variables were presented as number and percentage. For the comparison between groups, Student's *t*-test, Mann-Whitney *U*-test, or Fisher exact test were applied as appropriate.

The reproducibility of the method has already been assessed in the primary analysis, using the Bland Altman plot and calculating the Pearson correlation index (17).

Logistic regression models were used to evaluate the relationship between the lung area and death or hernia recurrence risk. Linear regression models were used to assess the effects of lung area on sPAP values. The models were corrected for gestational age at birth, as this variable could independently influence the lung development and survival of patients.

The ROC curve was also calculated to assess the discriminatory capacity of the radiographic measurement, thus analyzing the sensitivity and specificity of the test.

Statistical analysis was performed using IBM SPSS^®^Statistics V26.0. A *p*-value of 0.05 or lower was considered to be statistically significant.

### Data Availability

The manuscript illustrates the results of an exploratory secondary analysis of the principal study NeoAPACHE I, registered at the ClinicalTrials.gov with identifier NCT04396028. Datasets generated during and/or analyzed during the current study are available from the corresponding author.

## Results

**Figure 1:**
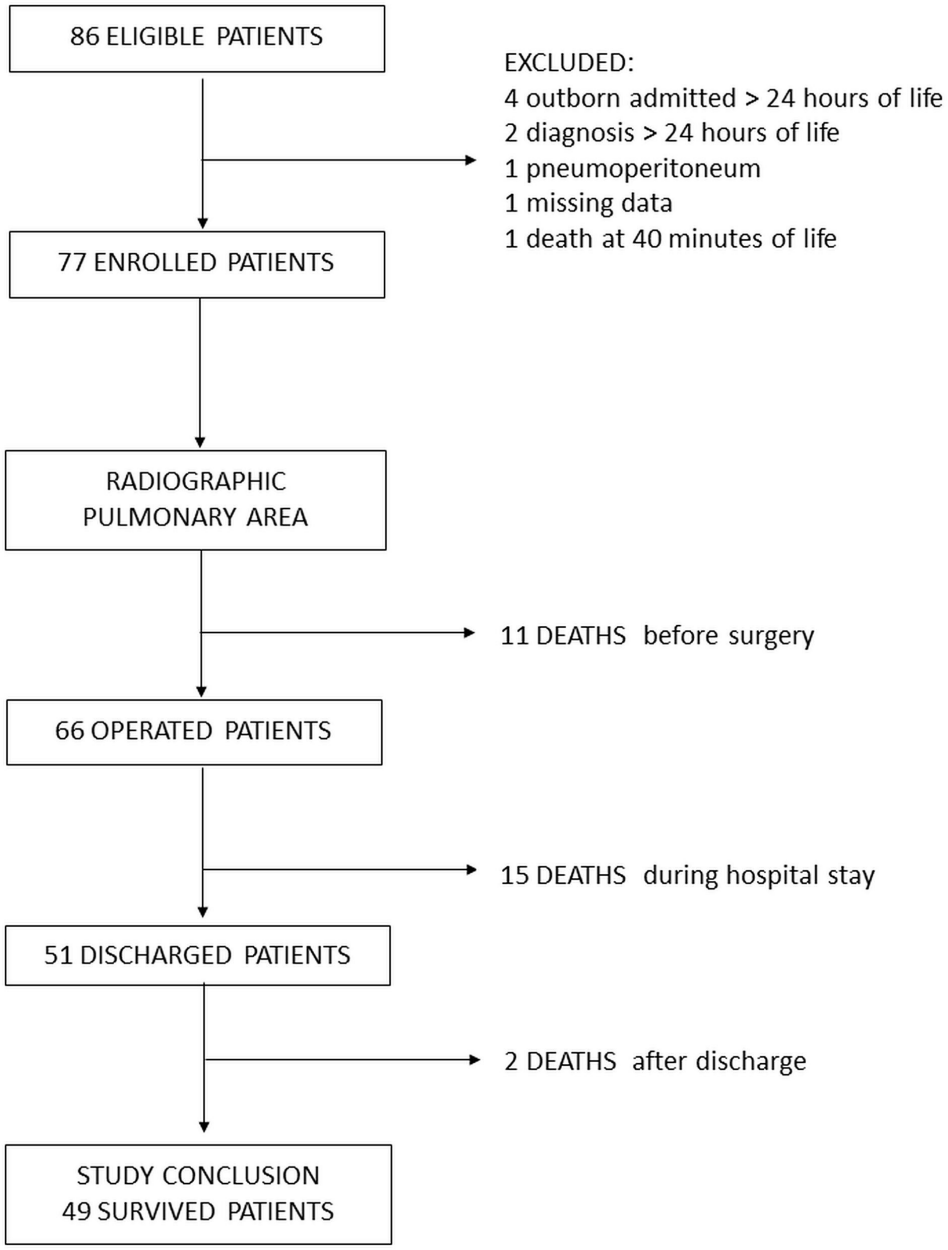
Study flowchart.

The radiographic pulmonary area was assessed on 77 patients, 49 of whom survived to discharge and were alive at the age of 1 year (36.4% mortality rate) (Figure 1). The majority of CDH were left-sided, with a high prevalence of severe forms and liver herniation. Fetal endoscopic tracheal occlusion (FETO) was performed in one-third of the cases, while extracorporeal membrane oxygenation (ECMO) was required in three patients. In more than half of cases, a diaphragmatic patch was needed for surgical repair, and in one patient, an abdominal patch was also used (Table 1).

**Table 1:** Characteristics of the study population.

**CDH (*****n*** **=** **77)**	
**Prenatal data**	
Side of defect—*n* (%) - Left CDH - Right CDH - Bilateral CDH	61 (79.2) 15 (19.5) 1 (1.3)
O/E LHR%—mean (*SD*) - Initial - Final	35.3 (12.7) 49.4 (15.7)
Liver up—*n* (%)	51 (66.2)
Grading CDH—*n* (%) -Severe - Moderate - Mild	32 (41.6) 13 (16.9) 32 (41.6)
FETO—*n* (%)	28 (36.4)
**Postnatal data**	
Gestational age (weeks)—mean (*SD*)	36.6 (2.2)
Birth weight (g)—mean (*SD*)	2744 (586)
Males—*n* (%)	43 (55.8)
Inborn—*n* (%)	74 (96.1)
Vaginal delivery—*n* (%)	40 (51.9)
APGAR 1 min—median (IQR)	6 (4–7)
APGAR 5 min—median (IQR)	8 (7–9)
Surgery—*n* (%)	66 (85.7)
Day of surgical repair—median (IQR)	3 (2–4)
Diaphragmatic patch (on operated)—*n* (%)	34 (51.5)
Abdominal patch (on operated)—*n* (%)	1 (1.5)
Mechanical ventilation (days)—median (IQR)	11 (7–20.5)
ECMO—*n* (%)	3 (3.9)
Length of stay (days)—median (IQR)	39 (15–68)
Deceased—*n* (%)	28 (36.4)
**Radiographic pulmonary area**	
Total pulmonary area (cm^2^)—mean (*SD*)	12.6 (7.0)
Ipsilateral pulmonary area (cm^2^)—mean (*SD*)	3.9 (3.5)
Contralateral pulmonary area (cm^2^)—mean (*SD*)	8.6 (4.3)

*CDH, congenital diaphragmatic hernia; ECMO, extracorporeal membrane oxygenation; FETO, fetal endoscopic tracheal occlusion; IQR, interquartile range; n: number; O/E LHR, observed/expected lung-to-head ratio; SD, standard deviation*.

### Radiographic Pulmonary Area, Pulmonary Hypertension, and Mortality

The study population was divided into two groups, deceased (*n* = 28) and survived (*n* = 49). Compared with survivors, deceased patients showed a lower mean observed/expected lung-to-head ratio (O/E-LHR%) both at diagnosis and before birth, and the liver was herniated more frequently. Moreover, both gestational age and weight were lower, and patch insertion was significantly higher (Table 2).

**Table 2:** Comparison between deceased and survived patients.

	**Deceased (*n* = 28)**	**Survived (*n* = 49)**	***p*-value**
**Prenatal data**			
Side of defect—*n* (%) - Left CDH - Right CDH - Bilateral CDH	22 (78.6) 6 (21.4) 0 (0.0)	39 (79.6) 9 (18.4) 1 (2.0)	0.856^∧^
O/E LHR%—mean (*SD*) - Initial - Final	28.4 (7.6) 42.1 (13.5)	40.1 (13.4) 54.2 (15.3)	<0.001^*^ 0.001^*^
Liver up—*n* (%)	28 (100)	23 (46.9)	<0.001^∧^
Grading CDH—*n* (%) - Severe - Moderate - Mild	19 (67.9) 7 (25.0) 2 (7.1)	13 (26.5)6 (12.2) 30 (61.2)	<0.001^∧^
FETO—*n* (%)	16 (57.1)	12 (24.5)	0.006^*^
**Postnatal data**			
Gestational age (weeks)—mean (*SD*)	35.6 (2.4)	37.2 (1.9)	0.002^*^
Birth weight (g)—mean (*SD*)	2437 (438)	2920 (591)	<0.001^*^
Males—*n* (%)	13 (46.4)	30 (61.2)	0.239^∧^
Vaginal delivery—*n* (%)	8 (28.6)	32 (65.3)	0.002^∧^
APGAR 1°min—median (IQR)	4.5 (3–6)	6 (5–8)	0.003°
APGAR 5°min—median (IQR)	7 (6–8)	8 (8–9)	<0.001^◦^
Surgery—*n* (%)	17 (60.7)	49 (100)	<0.001^∧^
Day of surgical repair—median (IQR)	3 (2–4)	2 (2–3.5)	0.603^◦^
Diaphragmatic patch (on operated)—*n* (%)	15 (88.2)	19 (38.8)	0.001^∧^
Abdominal patch (on operated)—*n* (%)	0 (0.0)	1 (2.0)	>0.999^∧^
Mechanical ventilation (days)—median (IQR)	8 (2–23.5)	16 (9–20)	0.036^◦^
Oxygen (days)—median (IQR)	7.5 (2–31)	13 (3–27)	0.426^◦^
Nitric oxide (days)—median (IQR)	8 (2–21)	9 (0–15)	0.380^◦^
Sildenafil (days)—median (IQR)	7 (2–29.5)	0 (0–31)	0.077^◦^
Length of stay (days)—median (IQR)	8 (2–31)	44 (35.5–70.5)	<0.001^◦^

*CDH, congenital diaphragmatic hernia; FETO, fetal endoscopic tracheal occlusion; IQR, interquartile range; n, number; O/E LHR, observed/expected lung-to-head ratio; SD, standard deviation*.

* *Student's T-Test*.

◦ *Mann Whitney U-Test*.

∧ *Fisher Exact Test*.

**Figure 2:**
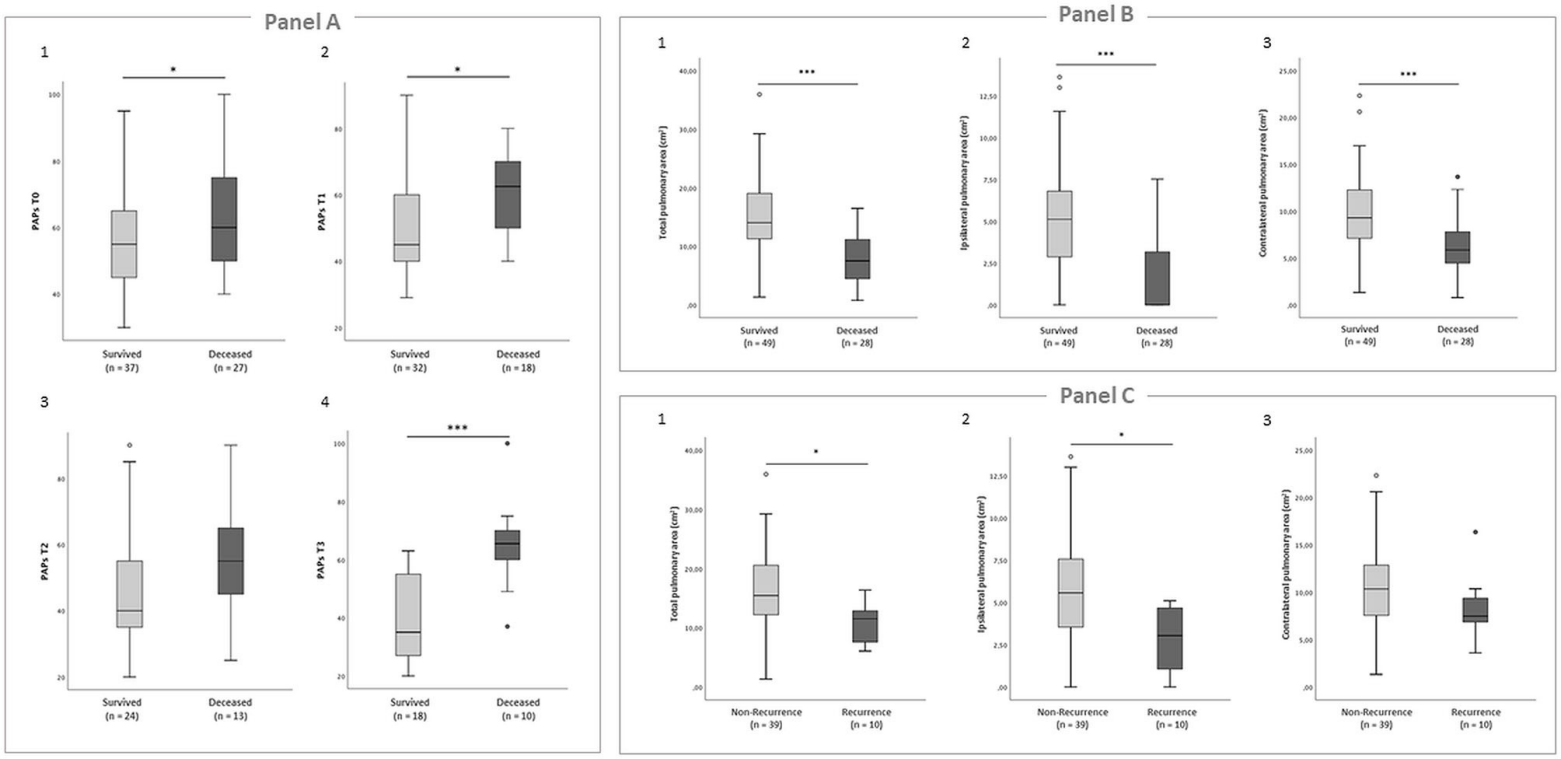
**(A)** Boxplots showing the comparison of sPAP values between survived and deceased patients. Student's *t*-test was performed to compare the two groups. (1) sPAP T0, *p* = 0.016; (2) sPAP T1, *p* = 0.022; (3) sPAP T2, *p* = 0.163; (4) sPAP T3, *p* < 0.001. **(B)** Boxplots showing the comparison of radiographic lung area on the first day of life between survived and deceased patients. Student's *t*-test was performed to compare the two groups. (1) Total pulmonary area, *p* < 0.001; (2) ipsilateral pulmonary area, *p* < 0.001; (3) contralateral pulmonary area, *p* < 0.001. **(C)** Boxplots showing the comparison of radiographic lung area on the first day of life between non-recurrence and recurrence patients. Student's *t*-test was performed to compare the two groups. (1) Total pulmonary area, *p* = 0.034; (2) ipsilateral pulmonary area, *p* = 0.011; (3) contralateral pulmonary area, *p* = 0.164.

At all times, deceased patients showed higher systolic pulmonary arterial pressure (sPAP) values compared with survivors (sPAP T0: 64.4 ± 17.2 vs. 54.4 ± 5.3 mmHg, *p* = 0.016; sPAP T1: 60.7 ± 10.9 vs. 50.6 ± 16.2 mmHg, *p* = 0.022; sPAP T2: 55.1 ± 17.9 vs. 46.4 ± 17.7 mmHg, *p* = 0.163; sPAP T3: 65.7 ± 16.5 vs. 38.8 ± 13.6 mmHg, *p* < 0.001; Figure 2A). They also showed lower mean total, ipsilateral, and contralateral pulmonary areas at birth (total pulmonary area: 8.1 ± 4.6 vs. 15.1 ± 6.9 cm^2^, *p* < 0.001; ipsilateral pulmonary area: 1.8 ± 2.6 vs. 5.1 ± 3.4 cm^2^, *p* < 0.001; contralateral pulmonary area, 6.2 ± 3.0 vs. 10.0 ± 4.3 cm^2^, *p* < 0.001; Figure 2B).

At birth, pulmonary area and sPAP were significantly associated: as the three areas increased, sPAP at T0 significantly decreased, as shown by the linear regression model (Table 3A).

**Table 3:** Radiographic lung area and outcome.

**A**	**Radiographic pulmonary area**	**sPAP at T0**		
	**Area (cm^**2**^)**	**Estimate**	**95%CI**	***p*-value**
	Total	−0.85	−1.44, −0.25	0.006
	Ispilateral	−1.84	−3.06, −0.62	0.004
	Contralateral	−1.09	−2.08, −0.09	0.032
**B**	**Radiographic pulmonary area**	**Death**		
	**Area (cm^**2**^)**	**OR**	**95%CI**	***p*-value**
	Total	0.78	0.69, 0.89	<0.001
	Ipsilateral	0.57	0.43, 0.76	<0.001
	Contralateral	0.76	0.63, 0.91	0.003
**C**	**Pulmonary hypertension**	**Death**		
	**sPAP (mmHg)**	**OR**	**95%CI**	***p*-value**
	T0	1.04	1.00, 1.07	0.034
**D**	**Radiographic pulmonary area**	**Recurrence**		
	**Area (cm^**2**^)**	**OR**	**95%CI**	***p*-value**
	Total	0.86	0.75, 1.00	0.042
	Ipsilateral	0.71	0.53, 0.95	0.022
	Contralateral	0.86	0.71, 1.05	0.148

*Results are corrected for gestational age. sPAP, Systolic Pulmonary Arterial Pressure*.

Following logistic regression analysis with death as the outcome variable, the increase in all radiographic parameters was also significantly related to improved survival in the first year of life (Table 3B).

Finally, with increasing sPAP at T0, the risk of death significantly increased as well (Table 3C).

**Figure 3:**
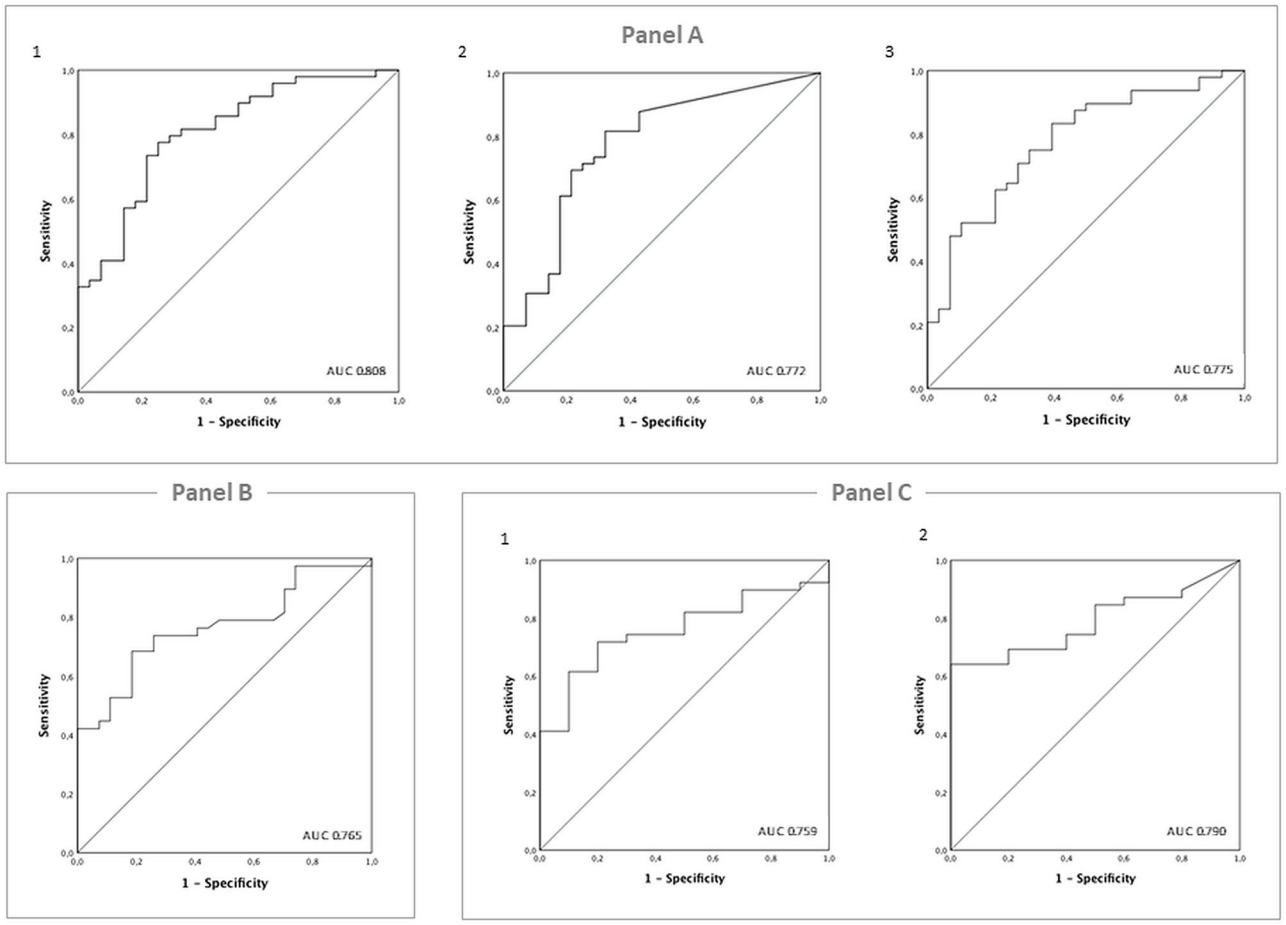
**(A)** ROC analysis and estimation of the corresponding area under the curve (AUC) for the radiographic pulmonary area's performance in predicting survival to 1 year of life. (1) Total pulmonary area; (2) ipsilateral pulmonary area; (3) contralateral pulmonary area. **(B)** ROC analysis and estimation of the corresponding area under the curve (AUC) for the O/E LHR% at diagnosis performance in predicting hernia recurrence in the first year of life. **(C)** ROC analysis and estimation of the corresponding area under the curve (AUC) for the radiographic performance of the pulmonary area in predicting hernia recurrence in the first year of life. (1) Total pulmonary area; (2) ipsilateral pulmonary area.

The receiver operating characteristic (ROC) curve analysis showed that the total pulmonary area had an area under the curve (AUC) of 0.808, and a cut-off of 10.87 cm^2^ predicted survival with 77.6% sensitivity and 75% specificity (Figure 3A1). The ipsilateral pulmonary area had an AUC of 0.772, and a cut-off of 2.08 cm^2^ predicted survival with 81.6% sensitivity and 68% specificity (Figure 3A2). The contralateral pulmonary area had an AUC of 0.775, and a cut-off of 7.3 cm^2^ predicted survival with 75% sensitivity and 68% specificity (Figure 3A3).

We finally performed aROC curve analysis using the O/E LHR at diagnosis. The total pulmonary area had an area AUC of 0.765, and a cut-off value of 31.7% showed 74% sensitivity and 74% specificity in predicting survival (Figure 3B).

### Radiographic Pulmonary Area and Hernia Recurrence

Survivors at the end of the first year of life were divided into two groups based on hernia recurrence: recurrence (*n* = 10) and non-recurrence (*n* = 39; Table 4).

**Table 4:** Comparison between recurrence and non-recurrence hernia patients.

	**Recurrence (*n* = 10)**	**Non-recurrence (*n* = 39)**	***p*-value**
**Prenatal data**			
Side of defect—*n* (%) - Left CDH - Right CDH - Bilateral CDH	8 (80.0) 2 (20.0) 0 (0.0)	31 (79.5) 7 (17.9) 1 (2.6)	>0.999^∧^
O/E LHR%—mean (*SD*) - Initial - Final	34.6 (8.2) 44.4 (14.6)	42.1 (14.4) 56.9 (14.6)	0.132^*^ 0.029^*^
Liver UP—*n* (%)	5 (50)	18 (46.2)	>0.999^∧^
Grading CDH—*n* (%) - Severe - Moderate - Mild	4 (40.0) 4 (40.0) 2 (20.0)	9 (23.1) 2 (5.1) 28 (71.8)	0.002^∧^
FETO—*n* (%)	3 (30.0)	9 (23.1)	0.690^∧^
**Postnatal data**			
Gestational age (weeks)—mean (*SD*)	37.5 (1.5)	37.1 (2.0)	0.563^*^
Birthweight (g)—mean (*SD*)	2808 (412)	2949 (630)	0.506^*^
Day of surgical repair—median (IQR)	3 (2.75–4.25)	2 (2–3)	0.066^◦^
Diaphragmatic patch—*n* (%)	6 (60.0)	13 (33.3)	0.156^∧^
Abdominal patch—*n* (%)	0 (0.0)	1 (2.6)	>0.999^∧^
Mechanical ventilation (days)—median (IQR)	20.5 (15.25–26)	12 (8–18)	0.013^◦^
Length of stay (days)—median (IQR)	55 (43–111.75)	42 (33–66)	0.028^◦^

*CDH, congenital diaphragmatic hernia; FETO, fetal endoscopic tracheal occlusion; IQR, interquartile range; O/E LHR, observed/expected lung-to-head ratio; SD, standard deviation*.

* *Student's T-Test*.

◦ *Mann Whitney U-Test*.

∧ *Fisher Exact Test*.

The recurrence group mainly included severe-moderate forms (80 vs. 28.2%), while most non-recurrence patients were mild (20 vs. 71.8%). Although the mean initial O/E-LHR% was not significantly different, the mean final O/E-LHR% was lower in the recurrence group (44.4 vs. 56.9%, *p* = 0.029). Even though diaphragmatic patching was higher in the recurrence group, this difference was not significant. Recurrence patients required longer intensive care (Table 4).

The mean total and ipsilateral pulmonary area were significantly lower in the recurrence compared with non-recurrence group (total pulmonary area: 11.0 ± 3.2 vs. 16.2 ± 7.2 cm^2^, *p* = 0.034; ipsilateral pulmonary area: 2.7 ± 2 vs. 5.7 ± 3.4 cm^2^, *p* = 0.011), while the mean contralateral area was not significantly different (8.3 ± 3.3 vs. 10.5 ± 4.5 cm^2^, *p* = 0.164; Figure 2C).

The logistic regression model showed that as the total and ipsilateral areas increased, CDH recurrence significantly decreased (Table 3D).

The ROC curve analysis showed that the total pulmonary area had an AUC of 0.759, and a cut-off of 13.07 cm^2^ predicted a 1-year follow-up free of hernia recurrence with 71.8% sensitivity and 80% specificity (Figure 3C1). The ipsilateral pulmonary area had an AUC of 0.790, and a cut-off of 3.75 cm^2^ had 74.4% sensitivity and 60% specificity (Figure 3C2).

### Comparison Between FETO and Non-FETO Patients

Mild cases have been excluded from the non-FETO population to achieve a more homogeneous CDH population of moderate-severe cases, either treated *in utero* or expectantly managed. The new population was constituted 45 patients, divided into 28 FETO (100% severe) and 17 non-FETO (76.5% moderate and 23.5% severe) (Table 5).

**Table 5:** Comparison between FETO and non-FETO patients.

	**FETO (*n* = 28)**	**Non-FETO, excluded mild (*n* = 17)**	***p*-value**
**Prenatal data**			
Side of defect—*n* (%)			
- Left CDH - Right CDH - Bilateral CDH	19 (67.9) 8 (28.6) 1 (3.6)	15 (88.2) 2 (11.8) 0 (0.0)	0.341^∧^
Liver UP—*n* (%)	28 (100)	13 (76.5)	0.016^∧^
O/E LHR%—mea *n* (SD)			
- Initial - Final	25.4 (5.6) 51.8 (15.4)	35.1 (7.9) 33.8 (7.1)	<0.001^*^ <0.001^*^
Grading CDH—*n* (%) - Severe - Moderate	28 (100) 0 (0.0)	4 (23.5) 13 (76.5)	<0.001^∧^
**Postnatal data**			
Gestational age (weeks)—mean (SD)	35 (2.4)	37.1 (1.7)	0.003^*^
Birthweight (g)—mean (*SD*)	2436 (511)	2517 (389)	0.576^*^
Surgery—*n* (%)	22 (78.6)	12 (70.6)	0.722^∧^
Day of surgical repair—median (IQR)	2.5 (2–3)	3.5 (2.25–5)	0.040^◦^
Diaphragmatic patch (on operated)—*n* (%)	18 (81.8)	8 (66.7)	0.410^∧^
Abdominal patch (on operated)—*n* (%)	1 (4.5)	0 (0.0)	>0.999^∧^
sPAP T0 (mmHg)—mean (*SD*)	59.4 (16.4)	63.2 (18.6)	0.513^*^
sPAP T1 (mmHg)—mean (*SD*)	55.2 (13.0)	63.1 (13.6)	0.136^*^
sPAP T2 (mmHg)—mean (*SD*)	48.3 (15.2)	53.5 (18.7)	0.459^*^
sPAP T3 (mmHg)—mean (*SD*)	54.6 (20.3)	42.9 (20.7)	0.228^*^
Mechanical ventilation (days)—median (IQR)	16 (9–25.5)	15 (5–23)	0.582^◦^
Oxygen (days)—median (IQR)	16 (4.75–34.75)	13 (2.5–45)	0.761^◦^
Nitric oxide (days)—median (IQR)	11.5 (6–22)	9 (2.5–17)	0.337^◦^
Sildenafil (days)—median (IQR)	9 (1–74.25)	6 (0–38)	0.334^◦^
Length of stay (days)—median (IQR)	41 (9–94.5)	39 (5–80.5)	0.512^◦^
Deceased—*n* (%)	16 (57.1)	10 (58.8)	>0.999^∧^
Recurrence (on survivors)—*n* (%)	3 (25.0)	5 (71.4)	0.074^∧^
**Radiographic pulmonary area**			
Total pulmonary area (cm^2^)—mean (*SD*)	10.5 (6.1)	8.9 (4.7)	0.362^*^
Ipsilateral pulmonary area (cm^2^)—mean (*SD*)	3.5 (3.2)	1.3 (2.0)	0.015^*^
Contralateral pulmonary area (cm^2^)—mean (*SD*)	6.9 (3.5)	7.6 (3.8)	0.523^*^

*CDH, congenital diaphragmatic hernia; FETO, fetal endoscopic tracheal occlusion; IQR, interquartile range; n, number; O/E LHR, observed/expected lung-to-head ratio; SD, standard deviation; sPAP, Systolic Pulmonary Arterial Pressure*.

* *Student's T-Test*.

◦ *Mann Whitney U-Test*.

∧ *Fisher Exact Test*.

FETO group was more severely affected, as showed by lower mean O/E-LHR% at diagnosis and a higher liver herniation rate. However, the mean O/E-LHR% before birth was higher.

No differences were found in the mean total and contralateral pulmonary area, while the mean ipsilateral pulmonary area was significantly increased in the FETO group.

Mean sPAP values, length of pharmacological treatments, and mechanical ventilation were not significantly different. Despite lower gestational age at birth, no significant difference in mortality rate was observed (FETO 57.1% vs. non-FETO 58.8%, *p* > 0.999). The recurrence rate among survivors did not reach statistical significance (*p* = 0.074).

## Discussion

Our study showed an association between radiographic lung area, sPAP values, and death, confirming pulmonary hypoplasia and pulmonary hypertension as the two most important determinants of mortality (25, 26, 36). Among survivors, lung area was also associated with hernia recurrence. As previously reported, our findings suggest a possible role of the radiographic lung area assessment as an easy, non-invasive, and reproducible tool in the early prediction of mortality and morbidity among patients with CDH (17, 18).

In our cohort, lower O/E-LHR% in the deceased group indicated a more severe fetal lung impairment, which was then reflected in smaller pulmonary areas at birth. Consequently, lung area and death were inversely related: 1 cm^2^ of rising in the ipsilateral area was associated with a 43% reduction in mortality, while variations in the total and contralateral area determined a reduction of 22 and 24%, respectively.

Wide defects have been previously associated with worse survival and pulmonary hypertension, suggesting that small lung size depicts the link between these two elements (6). Similarly, in our cohort, deceased infants were characterized by persistently higher sPAP values than survivors. In particular, sPAP values at birth showed a decreasing trend by 1.84 mmHg, with each 1 cm^2^ increase in the ipsilateral area.

Our findings were consistent with previous literature (13, 16, 18). A significantly lower CRTA was reported in newborns with CDH who died compared with survivors, and a CRTA >12.99 cm^2^ was found to predict survival to discharge from NICU better than LHR at diagnosis, with 85% sensitivity and 73% specificity (18). In our study, we considered the O/E LHR% at diagnosis instead of the absolute ratio, but similarly, the lung area performed better in predicting mortality.

After surgical repair, persistently elevated pulmonary pressure carried the highest mortality risk, with a 16% increase in death risk for each sPAP unitary increment. Several studies have correlated the severity of PH with mortality. Dillon et al. evaluated mortality in a cohort of CDH patients and reported that all those with supra-systemic sPAP died (26). Coughlin et al. reported that patients with higher pulmonary pressure at 1 month had a higher incidence of post-operative complications and worse survival, and persistently severe PH at 1 month was associated with increased mortality (6). Similarly, looking at our results, we could assume that the most critical factor might not be the absolute value of sPAP or the presence of PH in the first hours after birth, rather its persistence over time (6).

We also observed a significant association between preoperative radiographic measurements and hernia recurrence among survivors during the first year of life. The overall recurrence rate of 20.4% in our cohort was in line with the literature reports (19, 24, 37). In particular, the recurrence rate was higher in those patients with lower final O/E LHR%, prolonged invasive respiratory support, and need for intensive care. Similarly, Al-Iede et al. found a longer duration of mechanical ventilation and hospitalization in children with recurrence (21). Notably, these patients showed a significantly lower mean total pulmonary area at birth than non-recurrence, mainly due to a significantly lower ipsilateral pulmonary area.

As a consequence, we respectively, observed a 14 and 29% reduction in recurrence risk in our cohort per unit increment of the total and ipsilateral area. The total radiographic area had the best specificity in discriminating those patients at risk of recurrence, while the ipsilateral area showed better sensitivity. Taken together, the lower ipsilateral area and O/E-LHR% reflected the presence of a large diaphragmatic defect as the cause of poor lung development, indirectly confirming defect size as the leading risk factor for hernia recurrence (19–22, 24). In other words, we speculate that recurrence patients were somehow “predisposed” to this complication since birth and could have been identified early in the postnatal course. The recurrence group's high patching rate suggested the presence of a wide defect, although this difference did not reach statistical significance. We cannot deduce any specific contribution of the patch in determining the recurrence risk due to the low sample size.

We observed that tracheal occlusion improved lung development and outcome through the descriptive comparison between FETO and non-FETO patients' characteristics. Since prenatal treatment is reserved for severe cases of CDH, the FETO group included only patients at one end of the spectrum of disease severity (2). Nevertheless, final O/E-LHR% dramatically improved after the procedure, and the ipsilateral lung area at birth was even significantly better so that the total pulmonary area did not differ between the two groups. Likewise, Dassios et al. observed that patients previously submitted to the FETO procedure had a CRTA comparable with untreated patients with a similar mortality rate, reflecting a lung catch-up growth favored by the prenatal procedure (18).

In our cohort, the non-FETO group, which was primarily constituted by moderate cases, showed a 41.2% survival rate, in line with what is generally expected for this category of CDH (2, 38, 39). As observed by Doneè et al., tracheal occlusion allowed improved outcomes in the operative group, similar to a moderate population expectantly managed (40). Finally, the recurrence risk was not significantly different between the two populations, despite higher patching in the FETO group, as previously observed by Ali et al. (41). Although patch repair is a leading risk factor, the low recurrence rate suggests other factors besides patch use as possible re-herniation determinants (20, 42). Tsai et al. reported a non-significant difference in recurrence rate between patching and primary repair, despite a higher disease severity in the first group (20). Jawaid et al. reported a low incidence of recurrence in patients in which Gore-Tex^®^ patch was inserted (42). Although we cannot conclude on the patch's contribution to re-herniation, we can observe that lower radiographic area at birth could influence the risk of this complication and speculate that lung catch-up growth in FETO patients could confer the same recurrence risk as the untreated counterparts, which needs to be confirmed with further data (19, 21, 22, 24).

To the best of our knowledge, our study seems to be the first to evaluate the association between radiographic lung area and two important outcomes affecting newborns with CDH: pulmonary hypertension and hernia recurrence.

The radiographic measurement is easy, rapid, and can be performed soon after birth on the chest X-ray routinely performed at NICU admission. It would contribute to the early identification of infants at greater risk of developing higher sPAP values in the immediate postnatal period and a higher likelihood of long-term hernia recurrence and higher mortality. For example, the combined serial evaluation of lung area and sPAP over time could help to define trajectories related to the risk of persistently elevated sPAP and chronic pulmonary hypertension. Similarly, the preoperative radiographic assessment could help identify a subgroup of patients at higher risk of recurrence, directing them toward a tailored surgical follow-up.

The ipsilateral and contralateral areas were considered separately, evaluating the impact of hernia on each lung. We demonstrated that the ipsilateral area, which is more seriously affected by visceral herniation, has the most significant influence on patient outcomes.

Finally, focusing on FETO patients, we confirm the positive effects of the fetal procedure on lung catch-up growth and patient outcome.

Patients from our cohort showed a broad spectrum of disease severity, including infants requiring fetal surgery and ECMO support, and the standardization of treatment according to international guidelines guarantees uniformity of care.

A certain technical difficulty in tracing the lung perimeter in severe forms must be underlined as a limitation of the study. We arbitrarily decided to consider only those parts of the radiograms where a lung plot was present, corresponding to those regions effectively recruited and ventilated. However, the interference of mechanical compression exerted by the herniated organs plays a considerable role.

This methodological decision could constitute a bias leading to underestimating the lung dimensions since atelectasis areas had been excluded from the measurement. Therefore, after mechanical compression has been removed, the effective lung area evaluation could reliably define lung hypoplasia and associated outcome. For example, Dimitriou et al. calculated the difference between the pre- and post-operative radiographic measurements, showing that post-operative improvement was higher in patients with a good outcome. They concluded that poor prognosis was correlated to low post-operative rather than low preoperative values, which was probably more related to mechanical compression than lung hypoplasia (16). Therefore, the radiographic assessment of post-operative lung areas and the relative increase from preoperative values should be included in further analysis.

The neonatal ECMO Center was activated in 2016, with only three patients undergoing extracorporeal support during the study period. This could have had an impact on survival chances. In addition, team training and expertise plays a major role in favorable ECMO outcome. We expect a survival improvement in the very recent years, which we would like to confirm with additional analysis (43–45).

Another significant weakness is related to the retrospective design of the study, which limited the sample size. Some missing data regarding sPAP estimation could not be integrated with further hemodynamic assessments. Therefore, only an exploratory secondary analysis could be performed. Finally, we recognize that several factors could influence pulmonary vascular resistance and mortality throughout the hospital stay, such as pharmacological treatments, infections, patency of the ductus arteriosus, or surgery timing. Similarly, radiographic lung area could be influenced by factors such as quality of image, ventilator settings, under- or overinflation, which can then influence vascular resistance as well. The contribution of these factors cannot be completely assessed with single imaging performed at birth, which reflects the patient's conditions in a defining moment. Although the highest lung area can be considered a good approximation, it might not reflect the patient best clinical condition, and these factors should be taken into account in further analysis on a larger cohort. In addition, it would be of great importance to match lung area and sPAP values at T1, T2, and T3, to clarify if the association is still confirmed over time and define possible trajectories.

## Conclusions

The radiographic pulmonary area on the first day of life reflects impaired lung development during fetal life and the extent of the diaphragmatic defect in CDH patients. Lower lung areas are associated with higher sPAP values at birth, death, and hernia recurrence. Further studies are needed to consolidate these results and define the possible role of the radiographic lung area for early risk assessment, monitoring, and outcome prediction in newborns with CDH.

## Data Availability Statement

The datasets presented in this study can be found in online repositories. The names of the repository/repositories and accession number(s) can be found at: ClinicalTrials.gov n° NCT04396028.

## Ethics Statement

The studies involving human participants were reviewed and approved by Milan Area 2, Italy. Written informed consent from the participants' legal guardian/next of kin was not required to participate in this study in accordance with the national legislation and the institutional requirements.

## Author Contributions

IA, GC, GR, SGa, SGh, VC, GB, NPes, and FMo contributed to the study's conception and design. IA, GR, GC, VC, SGa, SGh, and FMa wrote the first draft of the manuscript. IA, NPes, and GC calculated the sample size. IA and NPes performed the statistical analysis. IA and IB assessed radiographic pulmonary areas. IB, NPer, IF, FMa, AC, MC, and FMo provided extensive critical revision. All authors contributed to the manuscript's critical revision and read and approved the submitted version.

## Conflict of Interest

The authors declare that the research was conducted in the absence of any commercial or financial relationships that could be construed as a potential conflict of interest.

## Abbreviations

AUC: area under the curve
CDH: congenital diaphragmatic hernia
CRTA: chest radiographic thoracic area
ECMO: extracorporeal membrane oxygenation
FETO: fetal endoscopic tracheal occlusion
NICU: neonatal intensive care unit
O/E LHR%: observed/expected lung-to-head ratio
PH: pulmonary hypertension
ROC: receiver operating characteristic
sPAP: systolic pulmonary artery pressure.
